# *Enteromorpha* polysaccharide and yeast glycoprotein mixture improves growth, antioxidant activity, serum lipid profile and regulates lipid metabolism in broiler chickens

**DOI:** 10.1016/j.psj.2022.102064

**Published:** 2022-07-26

**Authors:** Teketay Wassie, Bei Cheng, Tiantian Zhou, Lumin Gao, Zhuang Lu, Jianlin Wang, Bekalu Mulu, Mengistie Taye, Xin Wu

**Affiliations:** ⁎ Key Laboratory of Agro-ecological Processes in Subtropical Region, Institute of Subtropical Agriculture, Chinese Academy of Sciences; National Engineering Laboratory for Pollution Control and Waste Utilization in Livestock and Poultry Production; Hunan Provincial Engineering Research Center for Healthy Livestock and Poultry Production, Changsha, Hunan, 410125, China; † Tianjin Institute of Industrial Biotechnology, Chinese Academy of Sciences, Tianjin, 300308, China; ‡ Animal Production and Technology Department, College of Agriculture and Environmental Sciences, Bahir Dar University, Bahir Dar, Ethiopia; § The Hubei Provincial Key Laboratory of Yeast Function, Angel Yeast Co., Ltd, Yichang, 443003, China

**Keywords:** antioxidant activity, broiler, Enteromorpha polysaccharide, lipid metabolism, yeast glycoprotein

## Abstract

This study aimed to analyze the growth performance, antioxidant activity, serum lipid profile, meat quality, and lipid metabolism of broiler chickens fed mixtures containing *Enteromorpha* polysaccharide (**EP**) and yeast glycoprotein (**YG**). A total of 400 one-day-old broiler chickens were randomly divided into 4 treatment groups of 10 replicates with 10 birds each replicate. The dietary treatments consisted of the control group (fed basal diet), and diets supplemented with *Enteromorpha* polysaccharide (EP; 400 mg/kg), yeast glycoprotein (YG;400 mg/kg), and EP+YG (200 mg/kg EP + 200 mg/kg YG). Compared with the control group, EP+YG supplementation enhanced growth performance and significantly reduced (*P* < 0.05) serum total triglyceride (**TG**), cholesterol (**CHOL**), and low-density lipoprotein LDL levels, and increased high-density lipoprotein (**HDL**) levels. Besides, birds fed EP+YG supplemented diet exhibited higher (*P* < 0.05) serum catalase (**CAT**), total antioxidant capacity, superoxide dismutase (**SOD**), and lower malonaldehyde (**MDA**) activities, and upregulated expressions of related genes, such as nuclear factor-erythroid factor 2-related factor 2 (*NRF2*), *SOD1*, and glutathione peroxidase 4 *(GPX4)* in the liver and intestinal tissues than the control group. Interestingly, higher (*P* < 0.05) serum SOD and lower MDA contents were observed in the EP+YG group than in either EP or YG group, suggesting a synergetic effect. Breast meat from EP+YG supplemented group had significantly higher redness value (a*), and lower pH24, total saturated fatty acid profiles, C14:0, C16:0, C18:0 fatty acid, atherogenic index, and thrombogenicity index than meat from the control group (*P* < 0.05). Furthermore, the mRNA expressions of fatty acid synthesis genes were downregulated (*P* < 0.05), whereas lipid β-oxidation-related genes were upregulated (*P* < 0.05) in the liver of the EP+YG supplemented group than in the control group. Overall, our data suggest that dietary EP+YG inclusion may have a synergistic effect, and therefore improve growth performance, regulate serum biochemical indexes, enhance antioxidant activity, and modulate lipid metabolism in broilers, indicating that it is a potential feed additive for chickens.

## INTRODUCTION

There is increasing interest in the use of marine algae, plant extract, and yeast extracts as a growth promoter and health benefit due to the banning of antibiotics utilization as a feed additive. However, research is ongoing to find a potential feed additive that can effectively replace antibiotics. Apart from their health benefits, these feed additives may also have effect on meat quality and composition. Meat from chicken is considered to have a low content of fat and is rich in desirable nutrients that make it preferred by consumers (Cavani et al., 2009). As one component of meat, fatty acids are used to assess nutritional value and product quality, including the impact on consumer health and sensory attributes, and therefore, there has been an increased interest in recent years in ways to manipulate the fatty acid composition of meat. It has been reported that the liver is the main site for lipid metabolism in chicken, accounting for more than 90% of the total fatty acid de novo synthesis (Leveille et al., 1975). Thus, lipid metabolism in broilers chicken is linked with hepatic lipogenesis (Daval et al., 2000; Kersten, 2001). A growing body of evidence confirmed that different genes, such as sterol regulatory element-binding protein-1c (***SREBP1c***), peroxisome proliferator-activated receptor α (***PPARα***), acetyl-CoA carboxylase (***ACC***), fatty acid desaturase-1 (***FADS1***), stearoyl-CoA desaturase (***SCD***), carnitine palmitoyltransferase 1a (***CPT1***), and acyl-CoA oxidase 1 (***ACOX1***) are involved in the hepatic lipid metabolism (Rocchi and Auwerx, 2000; Song et al., 2010).

However, studies have shown that oxidative stress interferes with lipid metabolism and has also a devastating effect on DNA and protein structure (Ezraty et al., 2017). This oxidative stress happens when there is an imbalance between the scavenging capacity of antioxidants and the generation of free radicals (Kim and Kim, 2018; Domínguez et al., 2019). Therefore, natural antioxidant supplementation is crucial to improve lipid metabolism and the overall health of animals. In this milieu, studies have confirmed the antioxidant properties of Enteromorpha polysaccharide (Feng et al., 2020) and yeast extract polysaccharide (Wang et al., 2021).

*Enteromorpha (Ulva) prolifera* (*E. prolifera*) is marine algae that contain sulfated polysaccharides that could exert various activities such as antioxidant, immunomodulating, hypolipidemic, antitumor, antibacterial, antiviral, and anticancer activities (Feng et al., 2020, Kim et al., 2011b; Wassie et al., 2021a). Extensive studies have shown that dietary *Enteromorpha* polysaccharide (**EP**) improved production performance, egg quality, antioxidant capacity, and intestinal morphology of chickens (Guo et al., 2020; Liu et al., 2020; Liu et al., 2021; Zhao et al., 2021). Besides, EP has been found to regulate intestinal microbiota in chicken (Wassie et al., 2021b), mice (Zhang et al., 2018), and fecal microbiota in humans (Kong et al., 2016). Qiu et al. (2021) reported that supplementation of diet with EP enhances weight and differentially regulates the gene expression at the transcriptome level in the bursa of Fabricius of Arbor Acres chickens. Furthermore, our previous studies demonstrated that EP-zinc supplementation could improve intestinal health in piglets (Xie et al., 2021), and regulate amino acid and fatty acid metabolism in chicken (Wassie et al., 2022).

Yeast glycoprotein (**YG**) is the yeast cell wall extract that possesses polysaccharides (β-glucan and mannan oligosaccharide) and protein that plays a vital role in animals’ health and growth. A growing body of evidence has shown that mannan oligosaccharide (**MOS**) supplementation improved growth performance (Kim et al., 2011a; Soumeh et al., 2019), oxidative status, and intestinal morphology and barrier function of chickens (Baurhoo et al., 2009; Hutsko et al., 2016). In addition, MOS ameliorates heat stress-induced intestinal damages (Cheng et al., 2019) and attenuates *Escherichia coli-*induced intestinal inflammation and barrier dysfunction in broiler chickens (Wang et al., 2016).

Similarly, dietary supplementation with yeast β-glucans has been demonstrated to promote growth performance (Tian et al., 2016) and enhance gut health in chickens (Anwar et al., 2017). In addition, β-glucans inclusion has been shown to stimulate humoral and cell-mediated immune responses (Omara et al., 2021; Wang et al., 2021), alleviated aflatoxin B (1)-induced DNA damage in lymphocytes (Zimmermann et al., 2015), and prevented *C. perfringens*-induced necrotic enteritis (Tian et al., 2016), thereby used as an alternative to antibiotics (Schwartz and Vetvicka, 2021). Supplementation of broiler's diet with mannan-oligosaccharide and β-glucan could improve the immune response (Awaad et al., 2011; Fadl et al., 2020). Furthermore, our previous study indicated that supplementation of diet with yeast glycoprotein promotes growth performance and improves gut health in weaned piglets (Qin et al., 2019).

There is growing interest in using different active compounds together to exploit their synergistic effect. In this regard, Jen et al. (2021) extracted 2 polysaccharides having different molecular weights and compositions, and tested their combined effect. The first polysaccharide was glycan with high molecular weight and the second was a low molecular weight polysaccharide composed of glucose, mannose, and galactose. The authors reported that the combined treatment of these 2 polysaccharides exhibited synergistic effects on inhibiting pro-inflammatory mediator production. Similarly, Morán-Santibañez et al. (2016) evaluated the antiviral effects of polysaccharides from *Eisenia arborea* and *Solieria filiformis* and found that the combined polysaccharide exhibited the highest antiviral activities compared with the individual effect.

The bioactivity of polysaccharides is closely linked with their structure and composition*. Enteromorpha* polysaccharide is a sulfated polysaccharide composed of rhamnose (Rha), glucuronic acid (GlcA), glucose (Glc), galactose (Gal), and xylose (Xyl) whereas yeast glycoprotein contains mannan oligosaccharide and β-glucan. Although the individual effects of *Enteromorpha* polysaccharides, mannan oligosaccharides, and β-glucan on growth performance, antioxidant activity, and lipid metabolism have been extensively studied, their combined effect remains elusive. Given this, we hypothesized that combining these compounds may have a synergetic effect. Therefore, the purpose of this study was to investigate the combined effects of EP and YG on growth performance, serum biochemical indices, antioxidant status, and lipid metabolism of broiler chickens.

## MATERIALS AND METHODS

### Source of Enteromorpha Polysaccharide

The EP was produced from the marine algae *E. prolifera* and provided by Qingdao Seawin Biotechnology Group Co., Ltd. (Qingdao, China). The content of *Enteromorpha* polysaccharides (EP) was not less than 45%, and the molecular weight was 4,431 Da. The water-soluble sulfated polysaccharides of EP were extracted from the *E. prolifera* by an enzymatic method according to the procedure previously described (Chi et al., 2018). Briefly, the algae were washed with distilled water and dried at 60°C, then minced to get homogenate powder. The algal powders were soaked in water, and then the water extracts algae were subjected to stepwise enzymatic treatment with pectinase, cellulase, and papain at 50°C for 1:30 h. The enzyme reaction was then inactivated by heating the reaction at 90 to 100°C for 10 min, and then immediately cooled on an ice bath, centrifugal concentrated, ethanol precipitation, and finally spray drying to obtain the polysaccharide products (Lv et al., 2013). The monosaccharide composition was determined using high-performance liquid chromatography (**HPLC**) according to the procedure described by (Yu et al., 2017). Based on the HPLC analysis results, the monosaccharide composition of the *E. prolifera* polysaccharide used in this study was composed of rhamnose, glucuronic acid, glucose, galactose, and xylose with a molar percentage of 40.6%, 9.3%, 38.2%, 5.6%, and 6.3%, respectively.

### Birds Management

The experimental design and procedures used in this study were reviewed and approved by the Animal Care and Use Committee of the Institute of Subtropical Agriculture, Chinese Academy of Sciences. The animal experiments and sample collection strictly followed the relevant guidelines (2021-0036A).

A total of 400 healthy one-day-old male Ross-308 broiler chickens were obtained from a local hatchery and housed in wire cages (10 birds/cage) raised in a room where temperature and ventilation were controlled. The room temperature was kept at about 32°C for 3 d and gradually reduced by 1°C every other day until a temperature reached 24°C, and then maintained this temperature. Birds were kept in a room with a 23-h/1-h light/dark cycle throughout the experimental period. The light source used was incandescent bulbs at a light intensity of 30 lux. Chickens had access to ad libitum feed and water. All nutrients in experimental diets were formulated to meet or exceed the recommendations for broiler chickens (NRC, 1994). The dietary composition and nutrient levels of the basal diet are shown in Table 1**.**

**Table 1:** Ingredient composition and nutrient contents of basal diets.

Ingredients, %	1–21 d	22–42 d
Corn	55.41	57.64
Soybean meal, CP43%	31.00	27.30
Corn gluten meal	5.00	5.00
Soybean oil	3.60	5.60
Limestone	1.20	1.20
Dicalcium phosphate	2.00	1.60
_L_-Lysine	0.34	0.25
_DL_-Methionine	0.15	0.11
Premix^1^	1.00	1.00
Salt	0.30	0.30
Total	100.0	100.0
Nutrient levels, %		
Metabolizable energy, MJ/kg	12.53	13.18
Crude protein	21.09	19.60
Calcium	1.02	0.93
Available phosphorus	0.49	0.42
Lysine	1.20	1.06
Methionine	0.50	0.43
Methionine + cysteine	0.85	0.77
Arginine	1.35	1.23
Threonine	0.80	0.74

1 The premix provided per kilogram of diet: vitamin A, 15,600 IU; vitamin D3, 4,480 IU; vitamin E, 31 IU; vitamin B1, 2.4 mg; vitamin B2, 7.2 mg; vitamin B6, 6.3 mg; vitamin B12, 0.32 mg; niacin, 47 mg; pantothenic acid, 16.2 mg; folic acid, 1.6 mg; biotin, 0.26 mg; Cu, 10.4 mg; Fe, 75 mg; Zn, 71 mg; Mn, 83.1 mg; Se, 0.5 mg; I, 0.5 mg.

### Diet and Experimental Design

The experiment was performed in a complete randomized design, and 400 one-day-old male Ross-308 broiler chickens were randomly divided into 4 treatment groups of ten replicates with ten chickens per replicate. The first group was fed a basal diet (control group), and Group II to IV received a basal diet supplemented with 400 mg EP/kg diet (EP), 400 mg yeast glycoprotein/kg diet (YG), and 400 mg/kg diet (EP + YG in 1:1 ratio), respectively, according to the recommended dose by our previous study (Wassie et al., 2021b). The yeast glycoprotein was purchased from (Angel Yeast Co., Ltd, Hubei, China), and was composed of ≥ 12% mannan oligosaccharide, ≥ 12% β-glucan, and ≤ 35% crude protein. The experiment lasted for 42 d.

### Sample Collection

On d 42, blood samples were collected from one chicken from each replication (n = 10/treatment). The sera were separated by centrifuging at 4,000 rpm for 10 min at 4°C and stored at -20°C for subsequent analysis. The feed offers, leftover, and body weight were recorded to evaluate the growth performance. At the conclusion of the experiment (d 42), one chicken from each replication (n = 10/treatment) close to the average body weight of the group was humanely killed by cervical dislocation. Then, breast muscle, liver, and small intestinal tissues (jejunum and ileum) were isolated, immediately frozen in liquid nitrogen, and stored at -80°C for gene expression analysis.

### Determination of Serum Biochemical Index

The concentrations of serum total protein (**TP**), albumin (**ALB**), alanine aminotransferase (**ALT**), aspartate aminotransferase (**AST**) activities, glucose (GLU), cholesterol (**CHOL**), total triglyceride (**TG**), high-density lipoprotein (**HDL**), and low-density lipoprotein (**LDL**) were analyzed using automated Biochemistry Analyzer (Synchron CX Pro, Beckman Coulter, Fullerton, CA).

### Meat Quality

The pH value was determined from the breast meat using a portable pH meter (WTW pH 340i with a probe SenTix, Weilheim, Germany) at 45 min after slaughter and 24 h after 4°C storage. It was counted as pH 1 and pH 24, respectively. Measurement was taken 3 times, and the average value was calculated for each sample.

Color measurements of lightness (L*), redness (a*), and yellowness (b*) were carried out on the surface of the breast muscle after 24 h of storage at 4 °C. The measurement was undertaken at 3 random locations of each sample using a colorimeter (MiniScan EZ 4500L, HunterLab, Murnau), and the average was determined for each sample.

The cooking loss was determined using the method described by Honikel (1998). Briefly, 3 meat slices (3 × 5 cm) from the breast muscle of each sample were cut and weighed (initial weight). Approximately 80 g of meat slices were placed in an autoclave bag and cooked in a water bath until the center of the slice reached 70°C. After cooking, the meat samples were cooled in cold water, blotted dry, and weighed. Cooking loss was expressed as a percentage of the initial sample weight.

Drip loss measurement was carried out from breast muscle after 24 h. Approximately, 80 g of slices were placed in netting and suspended in an inflated bag at 4°C for 24 h. Thereafter, meat slices were weighed again, and drip loss was expressed as a percentage of the initial weight (Honikel, 1998).

### Serum Antioxidant Activity

The serum total antioxidant capacity (**T-AOC**, No. AK351), the total superoxide dismutase (**SOD**, No. AK061), and malondialdehyde (**MDA**, No. AK289) activity were determined using commercially available kits (Bioss, China) following the manufacturer's instructions. The content of catalase (**CAT,** No. ZC-S0615) was measured using a commercial kit (ZCi BIO, Shanghai, China) following the manufacturer's instructions.

### Fatty Acid Composition

The fatty acid composition was analyzed based on our previous study (Wassie et al., 2022). Briefly, the lipids were extracted from the breast muscle and quantified as methyl esters (FAME) using gas chromatography-flame ionization detection (Thermo Quest EC Instruments) with foil thickness of 50 m × 0.25 mm × 0.25 μm. The GC injection port temperature was set to 225°C in split mode (split ratio 50:1) using helium as the carrier gas at a constant flow rate of 1.2 mL/min. The detector temperature was set at 250°C, and the column temperature was 200°C. Individual fatty acid peaks were identified by comparing the retention times with the pure FAME standards run under the same operating conditions. The results are expressed as the percentage of the total identified fatty acids.

***Lipid Quality Ratios and Indexes***. The ratios of FA n-6 to n-3 (n-6/n-3), polyunsaturated fatty acid to saturated fatty acid (**P*/*S**) and atherogenic index (**AI**), and thrombogenicity index (**TI**) were calculated according to the proposed formula by Ulbricht (Ulbricht and Southgate, 1991).

n−6/n−3=Σn−6PUFA/Σn−3PUFA

P/S=ΣPUFA/ΣSFA

AI=[(4xC14:0)+C16:0]/[Σn−6PUFA+Σn−3PUFA+MUFA]

TI=(C14:0+C16:0+C18:0)/[(0.5xMUFA)+(0.5xΣn−6PUFA)+(3xΣn−3PUFA)+(Σn−3/Σn−6PUFA)]

Where, SFA: Saturated fatty acid; MUFA: Monounsaturated fatty acid; PUFA: polyunsaturated fatty acid.

### Quantitative Real-Time Polymerase Chain Reaction Analysis

Quantitative real-time polymerase chain reaction (qPCR) was used to investigate the effects of EP+YG supplementation on the expression of antioxidant and lipid metabolism-related genes. Briefly, total RNA was isolated from the frozen liver and intestinal tissues using a trizol reagent (Invitrogen Co., Carlsbad, CA) and then treated with DNase I (Invitrogen, Carlsbad, CA) according to the manufacturer instructions. The integrity was detected by 1% agarose gel electrophoresis, and the quality and quantity were assessed using Nanodrop 2000 (Thermo Fisher Scientific, Waltham, MA). The first-strand complementary DNA (cDNA) was then, synthesized using the Hifair II 1st strand cDNA synthesis kit (Shanghai Qianchen Biotechnology Company, Shanghai, China) according to the kit instruction. The RT-qPCR was performed on Roche LightCycler 480II (Roche, Basel, Switzerland) using SYBR Green mix (Takara, Tokyo, Japan) with targets and β-actin (housekeeping) genes primers (Table 2). The PCR reaction was run in triplicate, and relative gene expression levels were normalized to β-actin. Thermal cycling conditions were initial denaturation of 95°C for 30 s, followed by 40 amplification cycles of 95°C for 15 s, 60°C for 30 s, and 72°C for 60 s. The relative mRNA expression of genes was calculated using the 2^−ΔΔCt^ methods described previously (Livak and Schmittgen, 2001).

**Table 2:** Antioxidant-related and lipid metabolism-related genes’ primers used for a quantitative polymerase chain reaction.

Gene name	Accession No	Primer 5’ to 3’
Antioxidant related		
*NRF2*	NM_205117.1	F: GAGAAAGCCTTGCTGGCTCA
		R: TGAAGTATCTGTGCTCTGCGAA
*CAT*	NM_001030762.3	F: GCGCCCCGAACTATTATCCA
		R: ATACGTGCGCCATAGTCAGG
*SOD1*	NM_205064.1	F: GGCAATGTGACTGCAAAGGG
		R: ATGCAGTGTGGTCCGGTAAG
*SOD2*	NM_204211.1	F: TACAGCTCAGGTGTCGCTTC
		F: GCGAAGGAACCAAAGTCACG
*GPX1*	NM_001277853.2	F: TGCGCCCGATGTTTTCAAAG
		R: AACGTTACCCAGACTCACGG
*GPX4*	NM_001346448.1	F: GGGTGAAGTTCGACATGTTCAG
		R: GTTCCACTTGATGGCATTCCC
Lipid metabolism		
*ACC1*	NM_205505	F: AATGGCAGCTTTGGAGGTGT
		R: TCTGTTTGGGTGGGAGGTG
*SREBP1c*	AY029224	F: GCCCTCTGTGCCTTTGTCTTC
		R: ACTCAGCCATGATGCTTCTTCC
*PPARα*	AF163809	F: AGACACCCTTTCACCAGCATCC
		R: AACCCTTACAACCTTCACAAGCA
*SCD1*	NM_205064.1	F: TTGTCTGATGGAGATCATGGCTTC
		R: TGCTTGCCTTCAGGATTAAAGTGAG
*CPT-1*	AY675193	F: GGGACCTGAAACCAGAGAACG
		R: ACAGAGGAGGGCATAGAGGATG
*ACOX1*	NM_001012578	F: GCCAGGTGGACTTGGAAAGA
		R: GCTGCCGTATAGGAACAATGAAG
*β-actin*	NM 205518.1	F: ACCGGACTGTTACCAACACC
		R: CCTGAGTCAAGCGCCAAAAG

### Data Analysis

The data were analyzed using the statically analytical software (SAS 9.1 Institute, Inc., Cary, NC). The normality and homoscedasticity of the data variance were checked using the Shapiro-Wilk test and Levene's test, respectively. Then, data were subjected to one-way ANOVA using the GLM procedure, considering chicken as an experimental unit. The percentage data (cooking loss and drip loss) were analyzed after arcsine transformation. Statistically significant was considered when *P* < 0.05 and mean comparison was performed by Tukey test. The data are presented as the mean ± standard error of the mean (SEM).

## RESULTS

### Growth Performance

The growth performance results of birds fed a diet containing EP and YG alone or in combination are presented in Table 3. The results demonstrated that EP and EP+YG fed chickens had higher (*P* < 0.05) final body weight and average daily gain compared with chickens that were fed a basal diet, while YG-fed chickens showed no statistical difference. However, there were no significant differences in feed intake (**FI**) and feed conversion ratio (**FCR**) among the treatment groups.

**Table 3:** The growth performances of broiler chicken fed diet containing EP, YG, and EP+YG.

Parameters	Treatment groups				*P-*value
	Control	EP	YG	EP+YG	
IBW, g	41.14 ± 3.1	41.03 ± 2.6	41.23 ± 2.91	40.87 ± 2.64	0.95
FBW, g	2145.14 ± 32.01^b^	2243.11 ± 30.27^a^	2149.09 ± 29.37^b^	2223.99 ± 21.95^a^	0.036
ADG, g/d	50.10 ± 0.76^b^	52.43 ± 0.72^a^	50.19 ± 0.70^b^	51.97 ± 0.52^a^	0.038
FI, g/d	86.61 ± 1.94	87.40 ± 0.63	86.52 ± 1.64	88.91 ± 0.66	0.57
FCR	1.73 ± 0.02	1.67 ± 0.03	1.73 ± 0.04	1.71 ± 0.02	0.39

Abbreviations: ADG, average daily gain; FBW, final body weight; FCR, feed conversion ratio; FI, feed intake; IBW, initial body weight.

Data are presented mean ± SEM; n=10. Means across a row with different superscript letter denotes significant differences at *P* < 0.05.

### Serum Biochemical Index

To identify whether EP+YG supplementation influences the serum biochemical indices, sera collected on d 42 were analyzed using an automated biochemical analyzer (Table 4). Interestingly, birds fed a diet supplemented with EP+YG had significantly lower (*P* < 0.05) serum TG, CHOL, and LDL levels, and higher (*P* < 0.01) HDL levels compared with the control group. Furthermore, EP+YG supplementation reduced (*P* < 0.05) CHOL level compared with the EP group and increased (*P* < 0.01) HDL level compared with the YG group. Compared with the control group, EP supplementation significantly decreased serum TG levels and increased HDL levels (*P* < 0.05). Chicken-fed YG had lower (*P* < 0.05) CHOL and LDL levels compared with the control group. Dietary YG significantly decreased (*P* < 0.05) CHOL level compared with the EP group. In this study, the treatment group did not affect the serum concentration of TP, ALB, AST, ALT, and GLU.

**Table 4:** Serum biochemical indices of broiler chickens fed EP and YG diet individually and in combination (EP+YG) on d 42 of the experimental period.

Parameter	Treatment groups							*P*-value
	Control		EP		YG		EP+YG	
TP, g/L	34.75 ± 1.74	35.49 ± 1.82		32.79 ± 0.64		35.52 ± 2.24		0.64
ALB, g/L	16.00 ± 0.69	16.59 ± 1.06		15.15 ± 0.50		15.55 ± 0.85		0.62
ALT, U/L	2.52 ± 0.39	2.40 ± 0.59		2.64 ± 0.40		2.51 ± 0.41		0.99
AST, U/L	306.60 ± 17.50	304.20 ± 20.64		307.60 ± 16.03		280.90 ± 15.96		0.67
GLU, mmol/L	13.68 ± 0.73	14.47 ± 1.14		14.21 ±0 .43		14.13 ± 0.59		0.91
TG, mmol/L	0.52 ± .04^a^	0.42 ± 0.03^b^		0.47 ± 0.03^ab^		0.42 ± 0.03^b^		0.042
CHOL, mmol/L	3.62 ± 0.16^a^	3.59 ± 0.26^a^		2.93 ± 0.17^b^		3.00 ± 0.15^b^		0.018
LDL, mmol/L	0.76 ± 0.07^a^	0.73 ± 0.05^ab^		0.60 ± 0.04^b^		0.59 ± 0.05^b^		0.038
HDL, mmol/L	1.96 ± 0.09^b^	2.50 ± 0.18^a^		1.97 ± 0.11^b^		2.47 ± 0.10^a^		0.003

Data are presented as mean ± SEM; n = 10. Means with different superscript letter across a row indicates significant difference at *P* < 0.05. Abbreviations: ALB, albumin; ALT, alanine aminotransferase; AST, aspartate aminotransferase; CHOL, cholesterol; GLU, glucose; HDL, high-density lipoprotein; LDL, low-density lipoprotein; TG, triglyceride.

### Meat Quality

To assess whether EP+YG supplementation affected meat pH, we measured the pH of meat steak from breast muscle at 45 min (pH1) and 24 h (pH24) postmortem (Table 5). The EP and YG diet alone neither affect pH1 nor pH24, while EP+YG supplementation reduced (*P* < 0.05) pH24 compared with the control group. Besides, EP+YG supplementation increased (*P =* 0.002) breast meat redness value (a*) compared with the other groups. However, lightness (L*) and yellowness (b*), cooking loss, and drip loss were not influenced by the treatment groups.

**Table 5:** Meat quality attributes of broiler chicken fed diet containing EP, YG and EP+YG on d 42 of the experimental period.

Parameters	Treatment group				
	Control	EP	YG	EP+YG	*P* value
pH1	6.37 ± 0.16	6.39 ± 0.18	6.39 ± 0.16	6.36 ± 0.16	0.98
pH24	5.73 ± 0.05^a^	5.73 ± 0.09^a^	5.72 ± 0.11^a^	5.58 ± 0.16^b^	0.01
L*	56.82 ± 2.56	56.28 ± 2.39	57.56 ± 2.21	57.90 ± 2.26	0.42
a*	12.58 ± 1.67^b^	12.69 ± 1.1^b^	13.20 ± 0.88^b^	14.45 ± 1.64^a^	0.002
b*	17.83 ± 1.83	18.64 ± .27	18.35 ± 2.53	17.97 ± 1.83	0.83
Drip loss, %	2.53 ± 0.62	2.02 ± 0.71	2.05 ± 0.44	2.29 ± 0.42	0.16
Cooking loss, %	16.7 ± 1.26	17.39 ± 2.19	16.85 ± 1.06	16.43 ± 1.25	0.38

The value is presented as mean ± SEM form and means across a row bearing different superscript letters showed a significant difference at *P <* 0.05. L*: Lightness; a*; redness; b* yellowness.

### Serum Antioxidant Capacity

In the aim to evaluate whether EP+YG supplementation influences the antioxidant activities in chickens, we measured the serum antioxidant concentration from blood collected on d 42 of the experiment (Table 6). The results demonstrated that compared with the control group, supplementation of diet with EP+YG improved the antioxidant activity, which is reflected by significantly higher (*P* < 0.05) serum CAT, T-OAC, and SOD content, while remarkably lower (*P* < 0.05) MDA content. Similarly, compared with the control group, serum CAT content was significantly increased (*P* < 0.05), while MDA level was significantly reduced in the EP group. Chickens in the YG group had remarkably higher (*P* < 0.05) serum T-OAC levels than chickens in the control group. Interestingly, birds fed a diet containing EP+YG exhibited higher serum SOD and lower MDA levels than birds fed EP or YG groups supplemented diet (*P* < 0.05).

**Table 6:** The serum antioxidant level of broiler chicken fed diet containing EP, YG, and EP+YG on d 42 of the experimental period.

Parameters	Treatment groups				*P-*value
	Control	EP	YG	EP+YG	
CAT ((U/mL)	15.46 ± 1.21^b^	21.70 ± 1.57^a^	18.87 ± 1.79^ab^	23.03 ± 2.31^a^	0.022
T-OAC (U/mL)	15.02 ± 0.94^b^	16.54 ± 0.98^ab^	18.11 ± 1.05^a^	18.40 ± 1.01^a^	0.036
SOD (U/mL)	0.08 ± 0.004^b^	0.087 ± 0.005^b^	0.094 ± 0.009^b^	0.118 ± 0.011^a^	0.012
MDA (nmol/mL)	1.26 ± 0.30^c^	0.98 ± 0.21^b^	1.07 ± 0.31^bc^	0.59 ± 0.13^a^	0.042

Data are presented mean ± SEM; n = 10. Means with different superscript letter across a row indicates significant difference at *P* < 0.05.

CAT, catalase*;* MDA, malondialdehyde; SOD*,* superoxide dismutase; T-AOC, total antioxidant.

### Fatty Acid Composition

**Figure 1:**
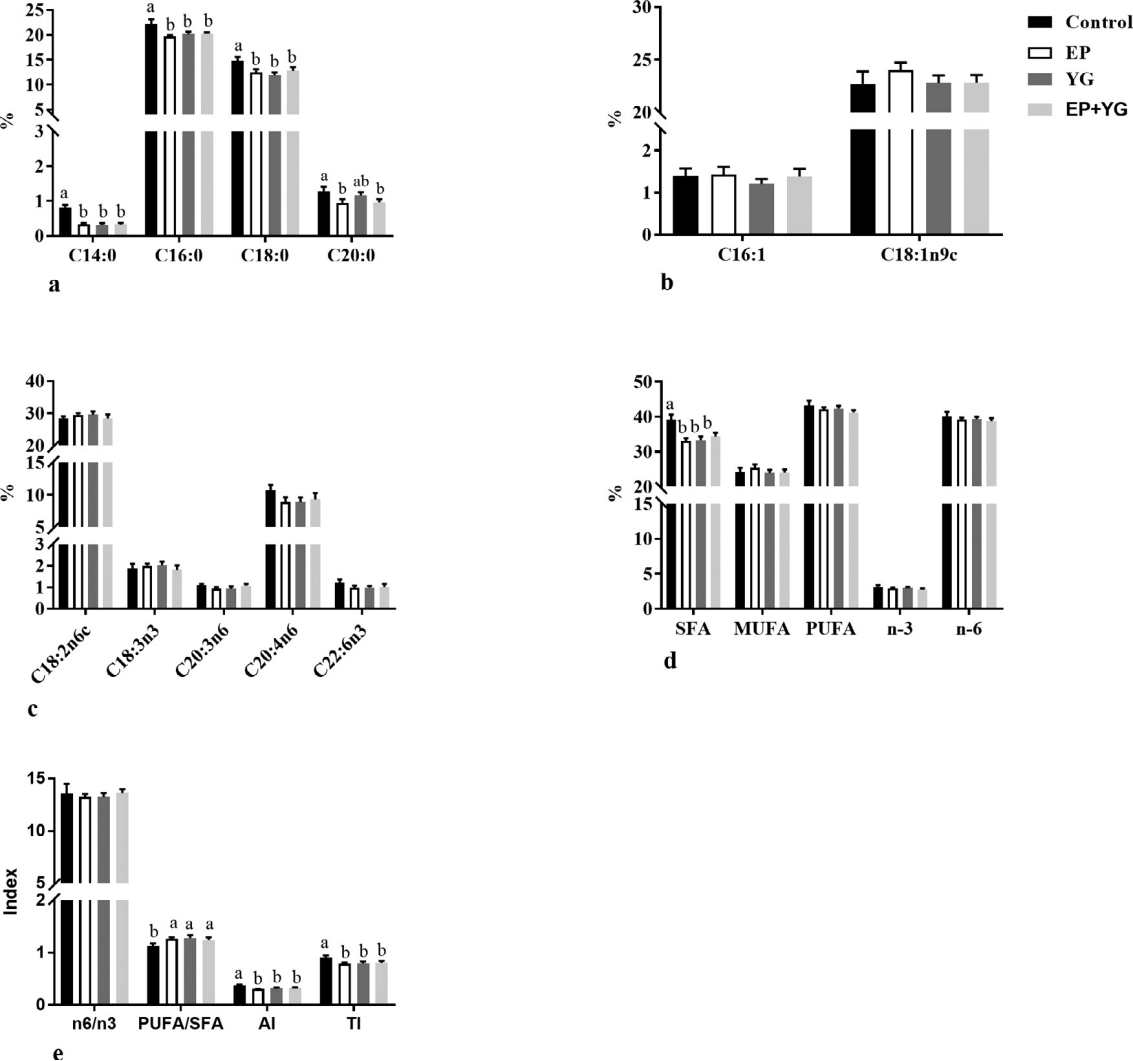
Effect of dietary EP, YG, and EP+YG supplementation on the breast muscle fatty acid composition of broiler chickens. Data are presented as mean ± SEM, n = 10. * and ** indicates statistical significances at *P* < 0.05 and *P* < 0.01, respectively by one-way ANOVA. Total SFA includes C14:0, C16:0, C18:0, and C20:0; total MUFA includes C16:1 and C18:1n9c; total PUFA includes C18:2n6c, C18:3n3, C20:3n6, C20:4n6, and C22:6n3; total PUFA n-6 includes C18:2n6c, C20:3n6, and C20:4n6; total PUFA n-3 includes C18:3n3 and C22:6n3. Abbreviations: AI, atherogenic index; MUFA, monounsaturated fatty acids; PUFA, polyunsaturated fatty acids; SFA, saturated fatty acids; TI, thrombogenicity index.

To evaluate whether dietary EP+YG supplementation regulates lipid metabolism, we determine the long-chain fatty acid profiles of breast muscle, and the results are presented in Figure 1. The data demonstrated that the majority of fatty acids observed in the breast muscle were C18:1cis9, C16:0, and C18:2n-6, which together accounted for about 76% of the total fatty acid. Dietary supplementation with EP and YG either individually or in combination significantly reduced (*P* < 0.05) the breast muscle total saturated fatty acid profiles and C14:0, C16:0, and C18:0 fatty acid. Breast meat from EP and EP+YG had significantly lower C20:0 content than the control group. Besides, a significant increment (*P* < 0.05) in the PUFA/SFA ratio and decrement (*P* < 0.05) in the AI and TI indices were observed in the supplemented groups compared with the control group. However, statistically significant differences among treatment groups were not detected in monounsaturated fatty acids, polyunsaturated fatty acids, Σn-3 PUFA, Σn-6 PUFA, and n6/n3 ratio.

### Hepatic and Intestinal Antioxidant Related Gene Expression

**Figure 2:**
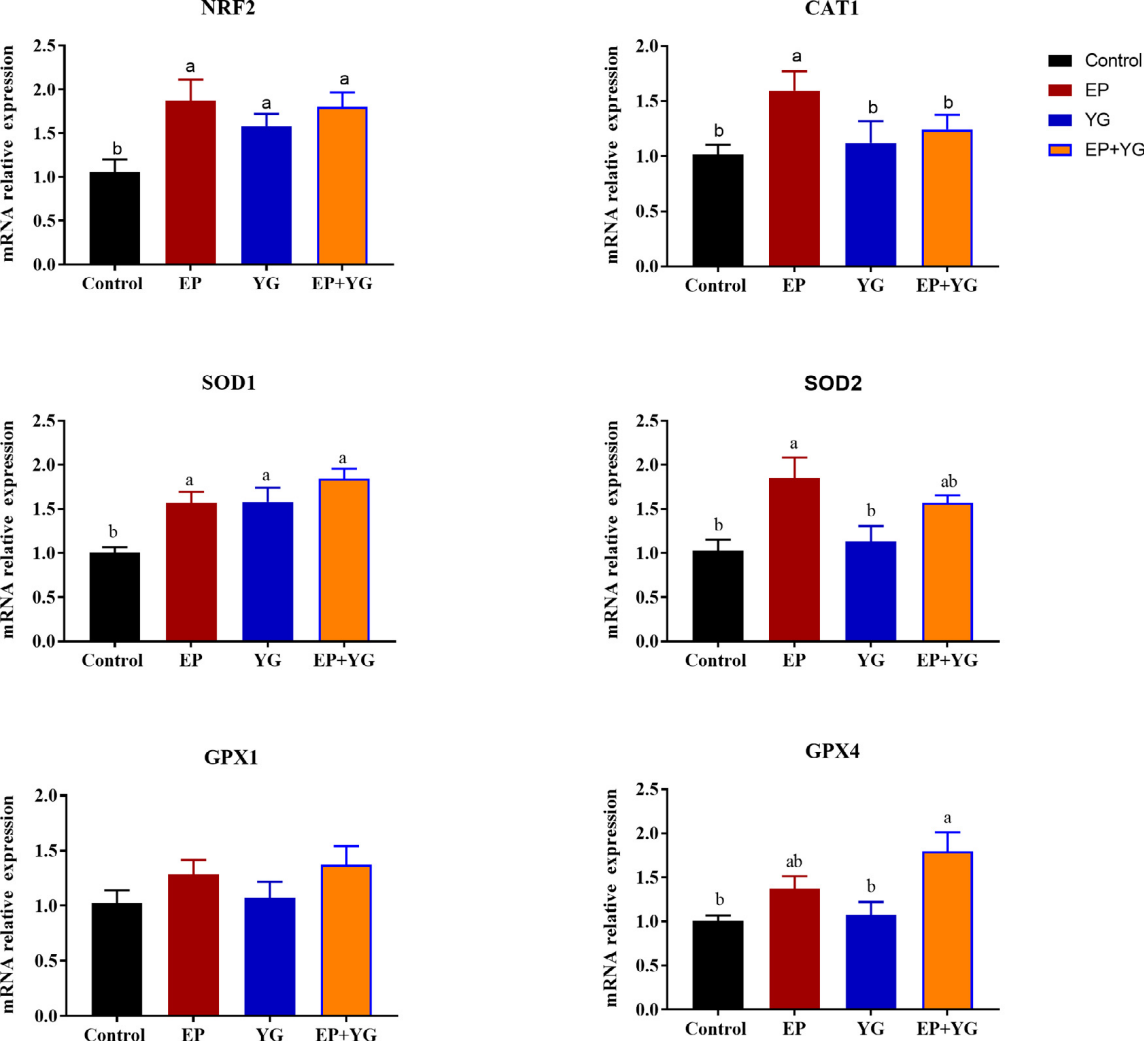
The mRNA expression of antioxidant-related genes in the liver tissues of broiler chickens fed EP and YG either individually or in combination. * and ** indicate significant differences at *P* < 0.05 and *P* < 0.01, respectively; n = 10.

To further insight into its effect on antioxidant activity, we detected the mRNA expression of genes related to antioxidants from the liver and intestinal tissues. The hepatic mRNA expressions of genes related to antioxidants are presented in Figure 2. In the liver, compared with the control group, dietary EP supplementation significantly upregulated (*P* < 0.05) the mRNA expression of nuclear factor-erythroid factor 2-related factor 2 (***NRF2***), *CAT, SOD1*, and *SOD2*. Likewise, YG supplementation significantly increased (*P* < 0.05) the expression of *NRF2* and *SOD1* genes compared with the control group. Interestingly, birds fed EP+YG had significantly higher expression (*P* < 0.05) of *NRF2, SOD1*, and glutathione peroxidase 4 (***GPX4***) compared with the control group and upregulated (*P* < 0.05) *GPX4* expression compared with the YG supplemented group.

**Figure 3:**
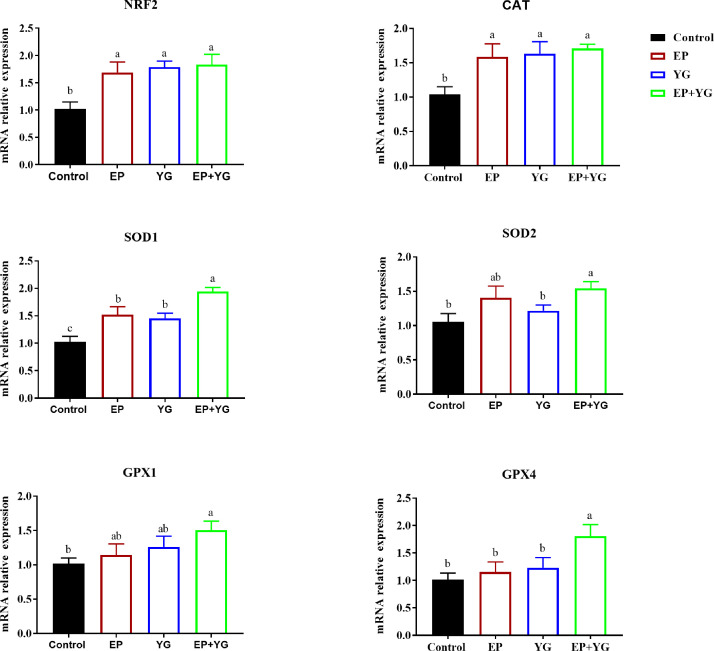
The mRNA expression of antioxidant-related genes in the jejunum of broiler chicken fed a diet containing EP and YG either individually or in combination. * and ** indicate significant difference at *P* < 0.05 and *P* < 0.01, respectively; n = 10.

In the jejunum, the inclusion of diet with EP and YG either individually or in combination significantly upregulated (*P* < 0.05) the mRNA expression of *NRF2, CAT*, and *SOD1* (Figure 3)**.** Compared with the control group, neither EP nor YG supplementation regulated the expression of *SOD2, GPX1*, and *GPX4* in the jejunum, but EP+YG addition markedly increased (*P* < 0.05) their expressions. Furthermore, birds fed a diet containing EP+YG had significantly higher (*P* < 0.05) mRNA expression of *SOD1* and *GPX4* than birds fed EP or YG supplemented diet, suggesting a synergetic effect.

**Figure 4:**
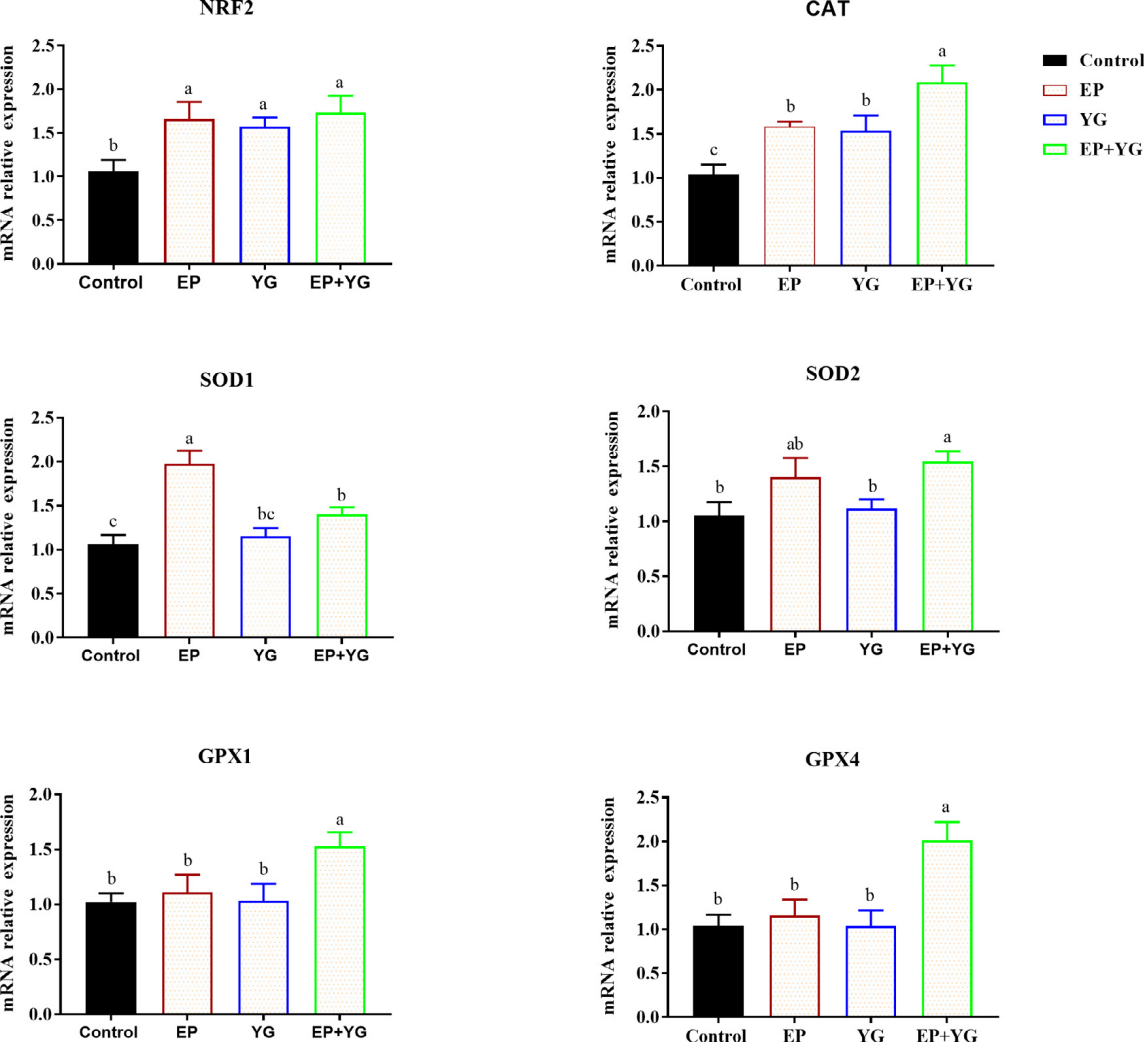
The individual and combination effect of dietary EP and YG inclusion on mRNA expression of antioxidant-related genes in the ileum of broiler chickens. * and ** indicate significant difference at *P* < 0.05 and *P* < 0.01, respectively; n = 10.

As indicated in Figure 4, in the ileum, the mRNA expression of *NRF2* and *CAT* (*P* < 0.05) was significantly upregulated in the supplemented groups compared with the control group. In addition, EP supplementation significantly increased the mRNA expression of *SOD1* compared with the control and YG supplemented group. Besides, neither EP nor YG supplementation regulated the expression of *SOD2, GPX1*, and *GPX4* in the ileum, however, EP+YG inclusion significantly upregulated (*P* < 0.05) their expressions compared with the control group.

### Hepatic Lipid Metabolism-Related Gene Expression

**Figure 5:**
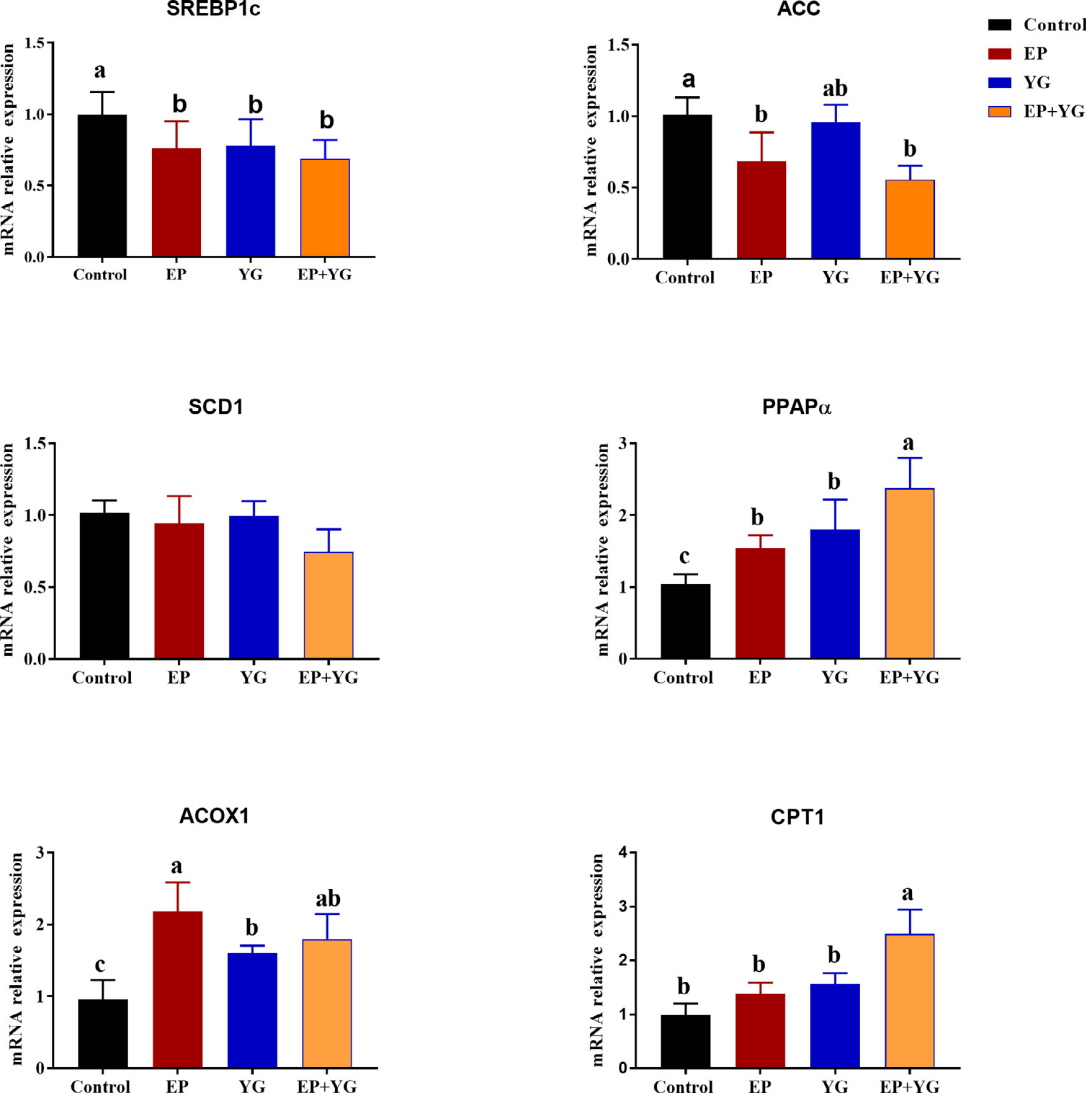
Effect of individually and in combination EP and YG supplementation on the lipid metabolism-related genes expression in the liver tissue of broiler chickens. Data are presented as mean ± SEM, n = 10. * and ** indicate significant differences at *P* < 0.05 and *P* < 0.01, respectively. Abbreviations: ACC, acetyl-CoA carboxylase; ACOX1, acyl-CoA oxidase-1; CPT1, carnitine palmitoyltransferase1a; PPARα, peroxisome proliferator-activated receptors alpha; SCD, stearoyl-CoA desaturase; SREBP-1c, sterol regulatory element-binding protein-1c.

Based on the results in the fatty acid composition, we further verified the mRNA expression of genes related to lipid metabolism (Figure 5). In this study, the mRNA expression of *SREBP1c* was significantly downregulated (*P* < 0.05), while the expressions of *PPAPα* and *ACOX1* were significantly upregulated (*P* < 0.05) in the supplemented groups than the control group. In addition, birds in EP and EP+YG supplemented group had lower expression (*P* < 0.05) of the *ACC* gene compared with the control birds. Furthermore, the expression of the *CPT1* gene was significantly upregulated (*P* < 0.05) in the EP+YG group than in the other groups. However, a significant difference in the expression of *SCD1* was not observed among the treatment groups.

## DISCUSSION

Feed additives have been reported to improve growth performance and health in chickens. In this study, we supplemented the broiler diet with EP and YG individually and in combination and evaluated their effect on growth performance, antioxidant activity, meat quality, and lipid metabolism. The results showed that the final body weight and ADG of chickens in the EP and EP+YG groups were higher than those of chickens in the YG and control groups. Consistent with the present study, the growth-promoting effects of algae-derived polysaccharides have been reported previously (Liu et al., 2020; Wassie et al., 2021b). Therefore, the increase in body weight in response to EP and EP+YG treatment observed in this study may be related to enhancement in nutrient absorption by improving gut morphology and function, supported by our previous study that showed EP treatment improved intestinal morphology by increasing villus height and reducing crept depth (Wassie et al., 2021b). However, a significant difference in growth performance was not observed between chickens fed YG supplemented diet and control diet. Similarly, Ahiwe et al. (2019) noted that yeast glycan and yeast mannan supplements did not affect body weight gain and feed conversion ratio in the starter phase in broiler chickens. Unlike the current study, Wang et al. (2021) reported that dietary yeast-derived products could improve average daily gain and feed efficiency in broiler chickens. The discrepancy between the current study with the previous study might be due to differences in dose, breed of chicken, and composition of the yeast product.

Dietary manipulation has been reported to influence meat quality (Belloir et al., 2019). Yet, studies are scant regarding the relationship between EP and YG supplementation on breast meat quality attributes. In this study, a lower pH value at 24 h (pH24) in the breast muscle of broilers fed EP+YG was observed compared with the control group. It has been reported that lower pH and water-holding capacity are associated with loss of soluble nutrients resulting in poor meat quality (Ozturk et al., 2012). However, the pH24 result obtained in this study was between 5.6 and 5.9, which is considered to be normal (Debut et al., 2003; Garcia et al., 2010), indicating that EP+YG supplementation did not negatively affect the meat quality. Furthermore, a significant effect of treatment on meat drip loss and cooking loss was not observed among treatment groups, indicating that neither EP nor YG and their combination did not affect water-binding capacity. Similarly, Wang et al. (2020) observed a nonsignificant effect of polysaccharide supplementation on drip loss of breast meat. Likewise, β glucan supplementation did not affect drip loss in chickens (Zhang et al., 2020). Color is an important attribute that influences consumer preference, and redness matches acceptability at purchase and is always favored by consumers (Wang et al., 2019). Although we did not observe a significant difference among treatment groups in terms of lightness (L*) and yellowness (b*), we found that breast meat from EP+YG supplemented chickens had a higher a* (redness) value than meat from the other groups, suggesting that EP+YG may improve color attributes in chicken meat.

Marine algae polysaccharides and yeast glycoproteins have been reported to reduce oxidative stress in broiler chickens (Cheng et al., 2019; Liu et al., 2020; Liu et al., 2021). Antioxidant enzymes, such as superoxide dismutase (SOD), catalase (CAT), and glutathione peroxidase (GPX) are produced by the body as a defensive mechanism to fight against the ROS (Cheng et al., 2014; Schieber and Chandel, 2014). In this study, compared with the control group, EP supplementation improved serum CAT content and reduced MDA levels, while dietary YG inclusion increased serum T-OAC levels suggesting the antioxidant role of EP and YG. Intriguingly, higher serum CAT, T-OAC, and SOD content, and lower MDA levels were observed in birds fed a diet containing EP+YG than in those fed only the basal diet, suggesting that EP and YG synergistically work to improve antioxidant activities. Oxidation of lipids by free radicals produced MDA (Gaweł et al., 2004), thus MDA content is a good indicator of detecting the potential antioxidant capacity of the body (Mishra and Jha, 2019). Similarly, improvements in antioxidant activities have been reported in chickens fed EP (Liu et al., 2020, Wassie et al., 2022), yeast β-glucans (Wang et al., 2021; Cheng et al., 2019).

To further understand whether the change in antioxidant activities in response to EP+YG was accompanied by a change in gene expression, we detected the mRNA expression of related genes. The current results showed that dietary supplementation of EP and YG individually or in combination improved the mRNA expression of *NRF2* and antioxidant-related genes, such as *CAT* and *SOD1* in the liver and small intestinal tissues, suggesting that the improvement in antioxidant activity might have resulted from the change in related gene expressions. Our results are consistent with previous studies that reported marine algae polysaccharides (Zhong et al., 2019), and yeast-derived products (Wang et al., 2021) regulated mRNA expression of antioxidant-related genes. In the jejunum and ileum, neither EP nor YG inclusion affected the mRNA expression of *SOD2, GPX1*, and *GPX4*, however, EP+YG supplementation upregulated their expression. This indicated that a blend of EP and YG might have a synergetic effect to improve antioxidant activity, which could be used as a potential natural antioxidant for broilers. Nuclear factor-erythroid factor 2-related factor 2 (*NRF2*) is a transcription factor that regulates the expression of antioxidant genes (Rajput et al., 2019). Normally, NRF2 binds to Kelch-like ECH-associated protein 1 (Keap1), is then rapidly degraded, and remains low in the cytoplasm. Upon oxidative stress, NRF2 exits from Keap1 and interacts with antioxidant response elements (AREs) in the nucleus, thereby activating the antioxidant-related genes and phase II detoxification enzymes (Keum, 2012). Therefore, the possible mechanism for improvement in antioxidant capacity observed in this study may be partly attributed to the modulation of the antioxidant signaling pathway, which previously explained that polysaccharides regulate the expression of the Keap1/NRF2-ARE signaling pathway (Sun et al., 2016).

Hyperlipidemia, a condition caused by abnormally high lipid levels in the blood, leads to heart disease and stroke (Jain et al., 2007). Enteromorpha polysaccharide and yeast glycan have been reported to perform hypolipidemic activities by reducing the serum TG, CHOL, and LDL levels (Korolenko et al., 2020). In this study, dietary supplementation of EP and YG, as well as their combination, improved serum lipid indices of broiler chicken. Dietary EP+YG inclusion significantly reduced serum TG, CHOL, and LDL levels, and increased HDL levels compared with the control group, suggesting a hypolipidemic activity. Interestingly, birds fed a diet containing the EP+YG had markedly lower CHOL levels compared with the EP group and higher HDL levels compared with the YG group. This indicated that EP+YG has a synergetic effect to improve the serum lipid indices, which might be a potential feed additive. The current result is consistent with previous studies that reported EP supplementation improves serum biochemical indices in chickens (Guo et al., 2021a, Wassie et al., 2021b) and rats (Tang et al., 2013). In line with our study, the hypolipidemic activities of mannan-oligosaccharide and β-glucan supplementation have been reported previously (Fadl et al., 2020; Korolenko et al., 2020).

It has been reported that diet could regulate the fatty acid composition of meat and thereby, influence the nutritional value and product quality, including consumer health and sensory attributes (Woods and Fearon, 2009). Herein, we analyzed the composition of fatty acid from the breast muscle of broilers fed a diet supplemented with EP and YG either individually or in combination (EP+YG). The results demonstrated that meat from supplemented chickens had lower saturated fatty acid content than meat from the control group. Saturated fatty acids are considered to have a detrimental effect on consumer health and are associated with metabolic disease, while MUFA and PUFA are reported to have a positive effect (Wood et al., 2004; Kennedy et al., 2009). Although we did not observe a significant difference among the treatment groups in the MUFA and PUFAs, the lower saturated fatty acid content in the supplemented groups suggested the beneficial effect of EP+YG supplementation on chickens’ meat value. In addition, AI and TI are important indicators of the health effect of food. Foods with higher AI and TI indices have a cholesterol-raising effect and stimulate cardiovascular disease (Krauss et al., 1996), whereas lower TI and AI indicate more beneficial effects of meat on consumer health (Ulbricht and Southgate, 1991). The present study demonstrated that dietary EP and YG inclusion either individually or in combination reduced the AI and TI indices in chicken meat, suggesting an improvement in meat quality. This effect might be associated with the decrease in saturated fatty acid in the supplemented group.

The liver is the primary site of fatty acid synthesis in poultry (Leveile et al., 1975). To study the molecular mechanism underlying the observed effects on the FA profile, we quantified the expressions of genes involved in lipid metabolism in the liver. In this study, the mRNA expression of *SREBP-1c* in birds fed EP*+*YG supplemented diet markedly decreased compared with the control birds fed basal diet, which is consistent with the result of decreased TG and CHOL in the serum of chickens. *SREBP-1c* is a transcriptional regulator of genes involved in fat syntheses, such as *ACC, FAS,* and *SCD*. It has been confirmed that targeted activation of *SREBP-1c* upregulated the expression of genes involved in FA and TG synthesis (Horton et al., 2002), whereas genetic ablation of *SREBP-1c* results in a downregulation in the expression of these genes (Liang et al., 2002). In addition, we found that EP+YG supplementation down-regulated the mRNA expression of the *ACC1*, a rate-limiting enzyme in de novo fatty acid synthesis that converts the acetyl-CoA to malonyl-CoA (Kim, 1997). This speculated that dietary EP+YG inclusion improved serum CHOL and TG levels via suppression of genes involved in de novo fatty acid synthesis. Consistent with the current study, previous studies have shown that EP inhibited the expressions of *SREBP-1c* and *ACC* and improved serum lipid profile in mice (Ren et al., 2018) and chicken (Wassie et al., 2022).

To further confirm the reduced fatty acid synthetase gene expression was accompanied by a change in fatty acid β-oxidation, we detected the mRNA expression levels of enzymes involved in the fatty acid catabolism in the liver tissue. Peroxisome proliferator-activated receptor α (*PPARα*) is a key transcription factor that regulates the expression of genes involved in fatty acid β oxidation such as *ACOX1* and *CPT1* (Muoio et al., 2002). The present study demonstrated that dietary EP and YG inclusion upregulated the expression of *PPARα* in the liver, but further increment was observed when EP and YG were supplemented together (EP+YG). This suggested that the EP+YG might have a synergetic effect to regulate fatty acid β oxidation through the *PPARα* gene. Furthermore, compared with the control group*,* EP+YG supplementation upregulated the hepatic expression of *ACOX1* and *CPT1* genes. Acyl-coenzyme A oxidase (*ACOX1*) is considered to be a rate-limiting enzyme in the peroxisomal β-oxidation pathway (Moreno-Fernandez et al., 2018). Carnitine palmitoyltransferase I (*CPT1*) catalyzes the transport of long-chain fatty acids into mitochondria for beta-oxidation (Weis et al., 1994). The increased hepatic lipolysis and decreased lipogenesis observed after EP and YG treatment may be due to the modulation of gut microbiota and microbiota-derived metabolites involved in lipid metabolism (Guo et al., 2021a). However, further metagenomics and metabolomics studies are needed to confirm.

## CONCLUSION

The inclusion of a diet with EP or YG improved antioxidant activities and serum biochemical indices but the higher improvement was observed when they were supplemented in combination (EP+YG), suggesting a synergetic effect. Besides, neither EP nor YG supplementation influenced meat quality, while EP+YG supplementation significantly reduced pH24 and increased breast meat redness value (a*) compared with the control group. Furthermore, dietary EP+YG supplementation enhanced growth performance and modulated lipid metabolism in broiler chickens. Taken together, this study demonstrated that dietary EP+YG inclusion could be a potential feed additive for chickens. This study provides insight into the application of different feed additives in combination to utilize their synergetic effect. Further 16S-rRNA sequencing and metabolomics studies are required to investigate the underlying mechanism of action.
